# Endophytic *Penicillium oxalicum* AUMC 14898 from *Opuntia ficus-indica*: A Novel Source of Tannic Acid Inhibiting Virulence and Quorum Sensing of Extensively Drug-Resistant *Pseudomonas aeruginosa*

**DOI:** 10.3390/ijms252011115

**Published:** 2024-10-16

**Authors:** Hoda S. Nouh, Nessma A. El-Zawawy, Mohamed Halawa, Ebrahim M. Shalamesh, Sameh Samir Ali, Grażyna Korbecka-Glinka, Awad Y. Shala, Shimaa El-Sapagh

**Affiliations:** 1 Botany and Microbiology Department, Faculty of Science, Tanta University, Tanta 31511, Egypt; nesma.elzawawi@science.tanta.edu.eg (N.A.E.-Z.); mohamad.lotfe2015@gmail.com (M.H.); ebrahim_shalamesh@science.tanta.edu.eg (E.M.S.); samh_samir@science.tanta.edu.eg (S.S.A.); shaymaa_elsabagh@science.tanta.edu.eg (S.E.-S.); 2 Department of Biotechnology and Plant Breeding, Institute of Soil Science and Plant Cultivation—State Research Institute, 24-100 Puławy, Poland; 3 Medicinal and Aromatic Plants Research Department, Horticulture Research Institute, Agricultural Research Center, Giza 12619, Egypt; awad.shala@yahoo.com

**Keywords:** endophyte, *Penicillium*, *Cactaceae*, tannic acid, multidrug-resistant bacteria, *Pseudomonas aeruginosa*, quorum sensing inhibitors

## Abstract

*Pseudomonas aeruginosa* is a harmful pathogen that causes a variety of acute and chronic infections through quorum sensing (QS) mechanisms. The increasing resistance of this bacterium to numerous antibiotics has created a demand for new medications that specifically target QS. Endophytes can be the source of compounds with antibacterial properties. This research is the first to examine tannic acid (TA) produced by endophytic fungus as a potential biotherapeutic agent. A novel endophytic fungal isolate identified as *Penicillium oxalicum* was derived from the cladodes of *Opuntia ficus-indica* (L.). The species identification for this isolate was confirmed through sequencing of the internal transcribed spacer region. The metabolites from the culture of this isolate were extracted using ethyl acetate, then separated and characterized using chromatographic methods. This led to the acquisition of TA, a compound that shows strong anti-QS and excellent antibacterial effects against extensively drug-resistant *P. aeruginosa* strains. Furthermore, it was shown that treating *P. aeruginosa* with the obtained TA reduced the secretion of virulence factors controlled by QS in a dose-dependent manner, indicating that TA inhibited the QS characteristics of *P. aeruginosa*. Simultaneously, TA significantly inhibited the expression of genes associated with QS, including *rhlR*/*I*, *lasR*/*I*, and *pqsR*. In addition, in silico virtual molecular docking showed that TA could efficiently bind to QS receptor proteins. Our results showed that *P. oxalicum* could be a new source of TA for the treatment of infections caused by extensively drug-resistant *P. aeruginosa*.

## 1. Introduction

Prickly pear (*Opuntia ficus-indica* L., Cactaceae) is one of the most widely distributed fruit crops in the world [1]. The nutritional properties, medicinal potential, and economic significance of fruits and cladodes have led to their long-standing use in traditional medicine [2]. Fruit peels and cladodes exhibit natural defenses against insects, germs, and other threats, suggesting they could be a valuable resource for developing novel medicines. Moreover, previous studies have shown that these parts of the prickly pear have antibacterial properties [3]. 

Natural compounds produced by microbes have contributed to significant advances in current therapeutics. Recent progress in microbiology has proven that endophytic fungi provide a wide variety of bioactive substances [4]. The majority of fungal endophytes are ascomycetes and imperfect fungi. They thrive within the host plant’s tissues without showing signs of illness, and they can influence important processes in the host’s physiology. Endophytes can improve plant growth, stimulate its defense against pathogens, and act as mediators in response to abiotic stress [5]. 

According to the WHO’s list of bacterial priority pathogens, *Pseudomonas aeruginosa* is classified as a high-priority pathogen that requires the urgent development of new drugs [6]. *P. aeruginosa* is a well-known opportunistic, invasive nosocomial, Gram-negative bacterium that causes a variety of acute and chronic infections, especially in the respiratory system [7]. Emerging resistance of this pathogen to multiple antibiotics has earned it global recognition as a serious threat. Multidrug-resistant and extensively drug-resistant (XDR) *P. aeruginosa* in lower respiratory tract infections significantly contribute to mortality on a worldwide scale [8]. The rapid increase in bacterial resistance, leading to a public health crisis, is attributed to the excessive use of antimicrobials.

Quorum sensing (QS) is a mechanism that controls bacterial virulence, pathogenicity, biofilm formation, and motility [9]. *P. aeruginosa* utilizes three QS signaling systems: rhl, las, and pqs. These systems communicate through cross-talk and interconnectedness, where each system is self-regulating and also influences the actions of the others. The auto-inducer for the las system is N-(3-oxo-dodecanoyl) homoserine lactone (3OC12 HSL), while the rhl system uses C4 (butanoyl) HS [10]. According to Schuster et al. [11], the expression of many genes encoding various virulence components is regulated by the las system. The third QS system, pqs, is controlled by an autoinducer called 2-heptyl-3-hydroxy-4(1H) quinolone (PQS). This autoinducer forms a complex with the transcriptional regulator PqsR, which then controls the activity of many target genes, including those involved in biofilm development. This mechanism eventually leads to the emergence of antimicrobial resistance and tolerance without requiring a specific enzyme to deactivate antibiotics [12].

Thus, the inhibitors of QS could have the double benefit of making the bacteria non-virulent and more susceptible to antibiotics. It is hypothesized that QS inhibitors could reduce pathogenicity because QS controls the synthesis of several virulence factors and contributes to the establishment of infection [13]. Therefore, compounds that have anti-QS activity could be valuable additions to the current treatments for *P. aeruginosa* infections [14]. It has been shown that in *P. aeruginosa*, taxifolin and naringenin decrease the levels of expression of genes that are associated with QS. Several other natural compounds with established biological features act as QS inhibitors by interfering with QS-related pathways, decreasing the expression of QS genes, and delaying the infection process [15,16]. 

**Figure 1:**
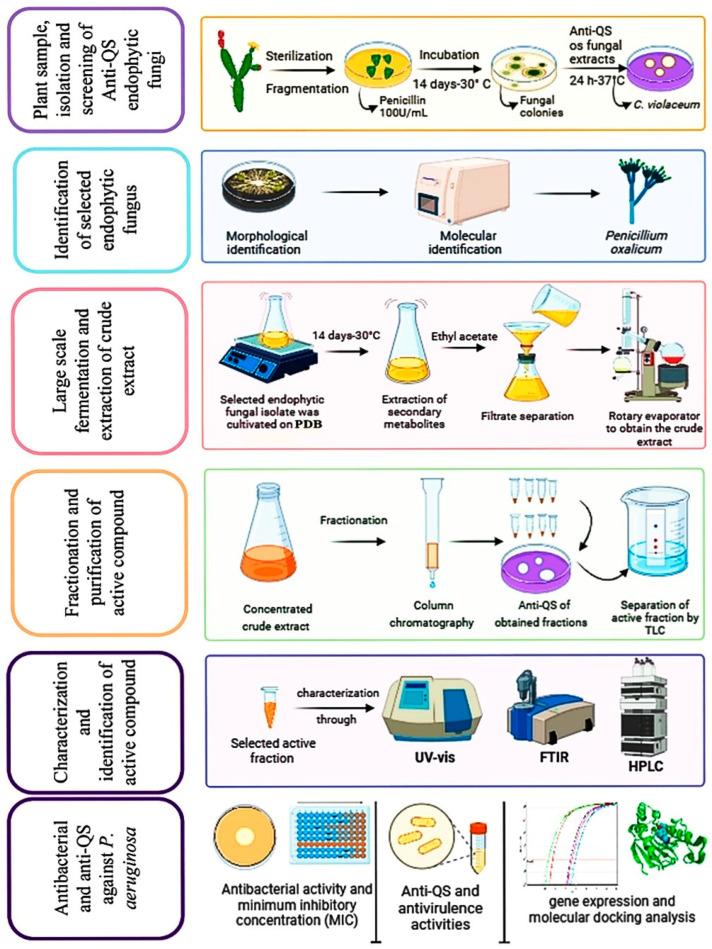
Experimental design used in this study.

Meena et al. [17] and Rashmi et al. [18] demonstrated that compounds derived from endophytic fungi isolated from *Carica papaya* also decrease QS activity in *P. aeruginosa*. Plant metabolites represent a valuable source of pharmacologically significant compounds that are beneficial for drug discovery [19]. However, utilizing medicinal plants to extract bioactive compounds comes with certain disadvantages, including dependence on seasons, loss of biodiversity, fluctuations in supply and demand, the endangered status of many medicinal plant species, and rising costs [20]. Biotechnological methods using various microorganisms provide promising alternatives for creating an unlimited, economical, and renewable reservoir of valuable bioactive products. Endophytic fungi associated with a medicinally important hosts can vary significantly in their capacity to produce a wide range of chemically potent and diverse secondary metabolites [21]. Therefore, the objective of our research was to isolate and identify the endophytic fungi from the cladodes of *Opuntia ficus-indica* L. and to determine the anti-QS and antibacterial properties of the fungal secondary metabolites against XDR *P. aeruginosa*. Subsequently, our study aimed to isolate, purify, and identify the most active compound responsible for the observed activity (Figure 1).

## 2. Results

### 2.1. Endophytic Penicillium oxalicum Isolate EF10 Showed the Highest Anti-QS Activity

Ten isolates of endophytic fungi were obtained from the cladodes of *Opuntia ficus-indica*. The isolates’ extracts were examined for their anti-QS activity against *Chromobacterium violaceum*. The isolate EF10 showed the highest activity with the diameter of the inhibition zone of violacein production of 23 mm (Table 1). Hence, it was selected as the most powerful isolate and then subjected to taxonomic identification based on morphological features and DNA sequencing. 

**Figure 2:**
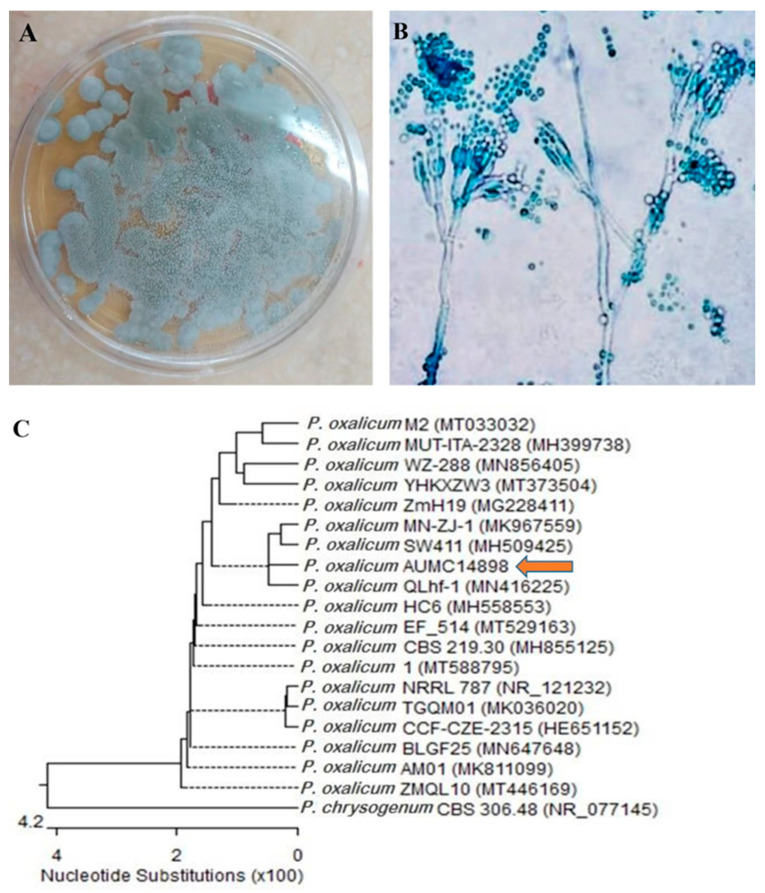
Morphological and molecular identification of *Penicillium oxalicum* AUMC 14898: (**A**) Colonies grown on potato dextrose agar at 30 °C for 7days. (**B**) Conidiophores and conidia at 40× magnification. (**C**) Phylogenetic tree based on ITS sequences of 18S rDNA, including the fungal strain isolated in this study (*Penicillium oxalicum* AUMC14898, arrowed).

The colonies of isolate EF10 exhibited a nearly flat morphology, characterized by a thin felt-like structure and a bluish-green color (Figure 2A). The mycelium exhibited conidiophores with broom-like branches, where ellipsoidal conidia were produced in chains at the apex of the pedicel (Figure 2B). The sequencing of the internal transcribed spacer (ITS) region and subsequent phylogenetic analysis confirmed the identification of this isolate as *Penicillium oxalicum* (Figure 2C). The isolate was labeled as AUMC 14898. The ITS sequence was deposited in GenBank under the accession number MZ025967. 

### 2.2. Tannic Acid as a Bioactive Compound-Inhibiting QS

Further, a bioactive compound was obtained from a crude extract of *P. oxalicum*. After obtaining the crude extract from the *P. oxalicum* culture, it underwent column chromatography using different solvent ratios. Eight fractions were collected separately and screened for their anti-QS activity. The most potent active fraction (F5), which showed the highest anti-QS activity (with 25mm inhibition zone of violacein production; Table S2) was then subjected to TLC fractionation. One band was observed on the TLC plate derived from a purified fraction and then scratched for further identification. The purified band from TLC was confirmed using UV spectrum analysis. The results revealed two peaks at 216 nm and 276 nm (Figure S2A), which were called the B-band of the benzenoid peak.

The FTIR spectra of the active fraction showed a strong absorption bands at 3403 and 2372 cm^−1^. The hydroxyl groups (OH) stretching vibrations are assigned to these bands. In this spectrum, an intense band is assigned to the C=O at 1629 cm^−1^. The spectrum of the benzene ring experiences a shift towards lower frequencies (1606 cm^−1^ and 1530 cm^−1^) as shown in Figure S2B. Moreover, a peak at 1345 cm^−1^ corresponds to the combined vibrations of the ring (C-C) and the carbonyl group (C=O), together with the involvement of the (C-H) and (C-OH) vibrations. Furthermore, the FTIR spectrum showed bands at 1178 cm^−1^, 1014 cm^−1^, and a broad band at 650 cm^−1^ which is related to the C=C. Thus, all the above functional groups confirmed the phenolic nature of the compound. The Raman spectrum of the active fraction showed a broad band at 1703 cm^−1^, which is related to the conjugated (C=O) vibration in the ester group (Figure S4A). This band may be due to several carbonyl groups and the presence of intra- and inter-molecular hydrogen bonds. A medium-intense band at 1611 cm^−1^ in the Raman spectrum is related to the C=C stretching. 

**Figure 3:**
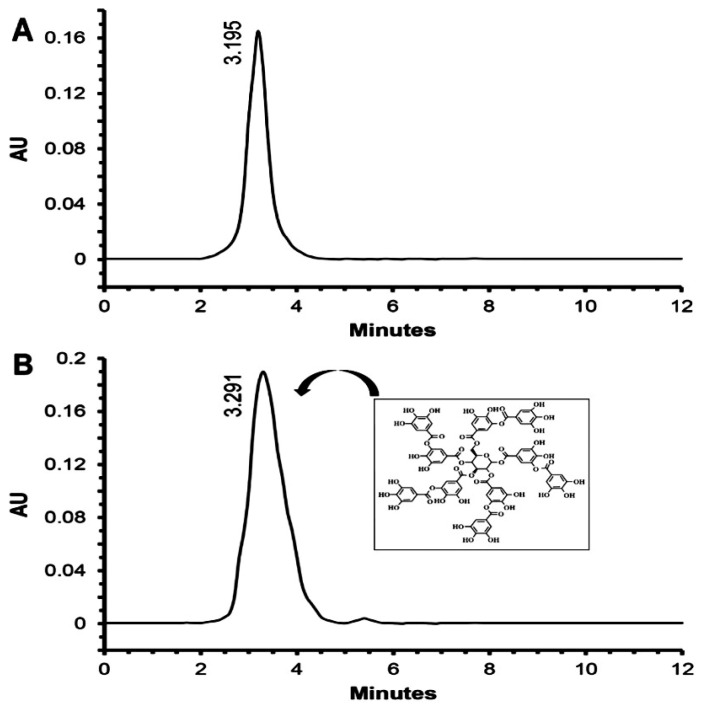
HPLC chromatogram of purified compound (**A**) and standard compound (**B**).

Moreover, ^1^H NMR data (Figure S4B,C) of the purified fraction revealed the presence of the anomeric protons of a glucose ring at δ range 4.11–6.15 ppm, aromatic protons at the region of δ 6.9 to 7.11 ppm, and OH groups at δ 9.27 and 10.09 ppm. The ^13^C NMR spectra showed the presence of glucose carbons at 68–92 ppm, aromatic carbons at 109–151 ppm, and remaining aromatic acid ester carbons at 164–168 ppm. These results revealed that the active fraction may be tannic acid (C_76_H_52_O_46_). Comparable spectra of the standard tannic acid gave similar results as presented in Figures S1 and S3. Finally, the HPLC fingerprint, as shown in Figure 3A,B, confirmed the active fraction as TA, which showed a major peak at the same retention time of standard tannic acid at 3.195 min. Based on our current knowledge, this study is the first to extract and purify TA from endophytic *P. oxalicum* isolated from *Opuntia ficus-indica*.

### 2.3. Inhibitory Activity of TA and MIC 

**Figure 4:**
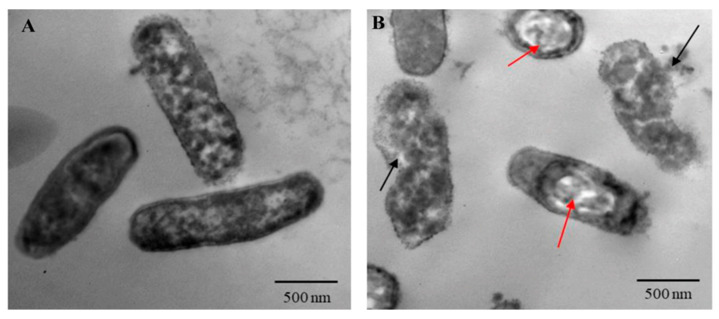
TEM images of *P. aeruginosa* PA-05 strain at 5000× magnification. (**A**) Untreated cells with intact cell membranes. (**B**) Treated cells with TA resulted in great morphological changes of the bacteria and lysis of cells (black arrows) in addition to development of vacuoles (red arrows).

As shown in Table 2, the purified TA showed antibacterial activity against all tested XDR *P. aeruginosa* isolates with variable inhibition zone diameters. (Comparable results were obtained for the standard TA; Table S3). PA-05 was the most susceptible isolate to TA and selected for further experiments. The MIC of TA was 125 µg/mL, while the MIC of baicalein (BCL) was 250 µg/mL (Figure S6). Moreover, antibacterial activity of TA against PA-05 was investigated by TEM imaging. Untreated bacteria showed uniform nuclear area and well dispersed cytoplasm with intact, well-defined rod-shaped cells (Figure 4A). Upon treatment with TA, bacteria demonstrated significant changes in their shape, beginning with the development of vacuoles within the cytoplasm, and the cell integrity was broken with releasing of cytoplasmic material to the surrounding environment (Figure 4B). Regarding growth curves, treating PA-05 strain with 100 µg/mL of TA inhibited cell density and growth, whereas 75 and 45 µg/mL had no impact on bacterial growth. On the other hand, both 200 and 175 µg/mL of BCL suppressed the bacterial growth; however 150 µg/mL did not affect the growth. Thus, the sub-MIC doses employed in this investigation were 75 µg/mL for TA and 150 µg/mL for BCL, as depicted in Figure S5.

### 2.4. Virulence Inhibition of PA-05 by TA 

**Figure 5:**
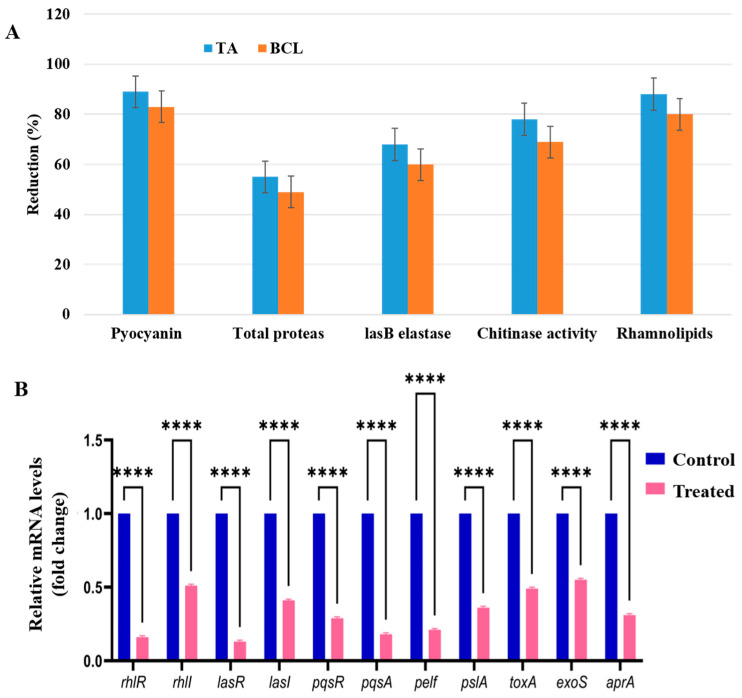
(**A**) Reduction of several virulence characteristics of *P. aeruginosa* PA-05 isolate by baicalein (BCL) and tannic acid (TA). (**B**) RT-qPCR analysis of various genes involved in quorum sensing (QS) of PA-05 isolate. The results are represented as ratios corresponding to the fold change of genes treated with tannic acid (TA) at sub-MIC value (75 µg/mL), as well as control (without treatment). For comparison of the experimental groups, two-way ANOVA was performed followed by Šídák’s multiple comparisons test. **** *p* < 0.0001.

Our results showed that TA inhibited the major virulence characteristics of PA-05 as a QS inhibitor. When PA-05 was subjected to the sub-MICs of TA and BCL, pyocyanin synthesis decreased by around 89 and 83%, respectively. The reduction of pyocyanin pigment is a good sign of RhlR/I pathway inhibition. Furthermore, the investigated substances had a considerable impact on the LasR/I-controlled elastase and proteases activity. TA and BCL inhibited Las B activity by 68 and 60%, respectively. TA and BCL both significantly decreased LasA protease activity, up to 55 and 49%, respectively. The chitinolytic activity of PA-05 was decreased by TA and BCL (78 and 69%, respectively). Around 88 and 80% of rhamnolipid inhibition was caused by TA and BCL in the PA-05 strain (Figure 5A).

### 2.5. TA Strongly Inhibits Motility of PA-05 

**Figure 6:**
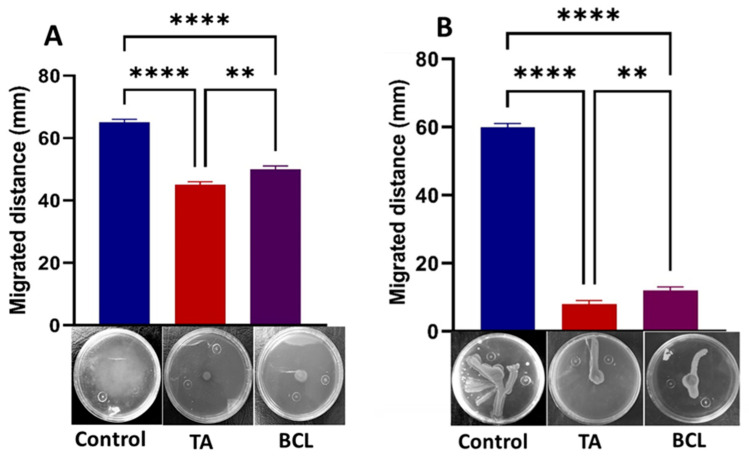
Effect of tannic acid (TA) and baicalein (BCL) at sub-MIC concentrations on motility of *P. aeruginosa* strain PA-05. Swimming (**A**) and swarming (**B**). Bar graphs show the average percentages of triplicate results. Values are mean ± standard error. For comparison of the experimental groups, one-way ANOVA was performed followed by Tukey’s multiple comparison test. ** *p*-value < 0.01 and **** *p* < 0.0001.

PA-05 flagella and type IV pili motility were likewise impacted by TA and BCL, indicating that these compounds had QS suppression and anti-biofilm characteristics. TA and BCL at sub-MICs had a deleterious effect on the motilities of PA-05, as it was observed that the mean swarming and swimming diameters were significantly less than those of the controls (Figure 6A,B).

### 2.6. TA Downregulates QS Genes and Its Relative Genes of PA-05

Validation of the impact of TA on virulence determinants at the transcriptional level was also conducted. The main QS genes of the Las, Rhl, and Pqs networks experienced transcriptional-level alterations. A number of genes regulated by QS that encode for virulence and biofilm activities, together with their crucial QS pathway genes, including *lasI*, *lasR*, *rhlI*, *rhlR*, and *pqsR*, were effectively suppressed. The genes responsible for encoding different extracellular virulence and biofilm components, including *aprA*, *pelA*, *pslA, exoS*, and *toxA*, were also impacted. The genes with the most significant decrease in expression were *lasR* (fold reduction of 0.13), *rhlR* (fold reduction of 0.16), and *pqsA* (fold reduction of 0.18). The gene expression levels of *rhlI*, *lasI*, *exoS*, and *toxA* showed moderate downregulation (Figure 5B).

### 2.7. Computational Investigations of the Interactions of LasR, RhlR, and PqsR with TA

**Figure 7:**
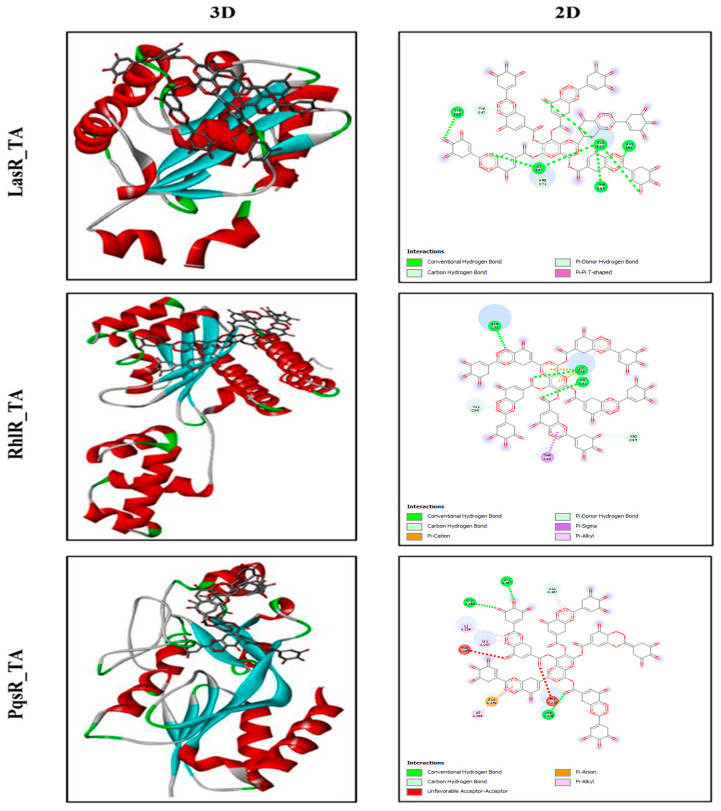
Molecular docking of tannic acid with LasR, RhlR and PqsR proteins represented in 3D and 2D conformations.

The biological interaction between TA and QS receptors (LasR, RhlR, and PqsR) was investigated through molecular docking (Figure 7). The obtained results revealed that TA binds strongly to all receptors. The highest docking score was observed for PqsR (−11 kal/mol), followed by LasR (−9.5 kal/mol) and RhlR (−9.2 kal/mol). TA formed four hydrogen bonds with the amino acids (GLN194, LEU197, ASN20, and ALA187) at the active site and had many hydrophobic interactions in the binding pocket residues of the PqsR protein. In the case of LasR, TA formed seven hydrogen bonds with TYR69, GLN81, THR95, LYS97, GLN98, ARG71, and TYR47 while creating four hydrogen bonds with HIS61, LYS66, GLN73, and VAL6 of the RhlR protein. The docking score, hydrogen bond length, and hydrogen bonds with receptors are summed in Table S4.

## 3. Discussion

Multidrug-resistant and extensively drug resistant (XDR) *P. aeruginosa* strains are emerging as major causes of death worldwide due to lower respiratory tract infections [8]. They are not only more severe, but their treatment often takes longer and requires more effort. Molecular shifts, enzyme-catalyzed medication degradation, cytosolic antimicrobial efflux, and changes in antibiotic permeability are some of the mechanisms behind microbial resistance to antibiotics [22]. Moreover, *P. aeruginosa* uses a signaling mechanism called quorum sensing (QS), which controls different physiological processes [23]. These processes include virulence, pathogenicity, biofilm formation, swimming, and swarming. The importance of QS in bacterial persistence and pathogenicity has made it a potential target for alternative anti-microbial therapies. However, little progress has been achieved in the field of QS-based alternative antimicrobial treatments. Instead, there is a growing trend toward reevaluating previously used medications, and numerous new classes of antibiotics have already been found [24]. Traditional medicinal herbs are predicted to provide more effective antibiotics. Multiple approaches have been utilized to discover new antimicrobial agents from the endophytic fungi inhabiting these plants [25] in order to enhance the action of current antibacterial medications. Endophytes, and fungi in particular, are crucial because of their capacity to produce bioactive compounds that defend plants from various pathogens [26].

Our research is the first to isolate *P. oxalicum* as an endophytic fungus from the *Opuntia ficus-indica* cladodes. The principal aim was to separate active metabolites from naturally occurring microbial sources. *Opuntia ficus-indica* has been utilized for hundreds of years to treat microbial infections, and antibacterial properties have been the subject of extensive study [3,8,27,28]. In addition, *Pencillium* is well recognized as a source of bioactive secondary metabolites [29,30] with shown antibacterial activity. A wide range of Gram-negative and Gram-positive pathogenic microbes, such as *P. aeruginosa*, *Escherichia coli*, *Staphylococcus aureus*, and *Bacillus* sp., were shown to be susceptible to the secondary metabolites isolated from different *Pencillium* species [31,32,33]. In this study, the crude extract of *P. oxalicum* was fractionated and purified, and the most active fraction with the greatest QS activity was identified as tannic acid. Our results were similar to those obtained by Espina et al. [34], who isolated and identified tannic acid from oat galls. However, to our knowledge, our study is the first report of tannic acid being isolated from the endophytic fungus *P. oxalicum* obtained from *Opuntia ficus-indica* cladodes.

The expression of *C. violaceum*’s pigment, violacein, is controlled by QS. As a result, inhibiting pigment synthesis provides a visual way to identify any *C. violaceum* inhibitor. Therefore, it has been employed as a reporter model in QS investigations as a marker feature [35]. Tannic acid was determined to be the component of the fraction with the highest anti-QS activity, with a 25 mm inhibition zone of violacein pigment. This effect is stronger than that in previous research, in which two phenethylamide compounds obtained from marine bacteria *Halobacillus salinus* were shown to limit violacein synthesis with inhibition zones of 10 mm and 13 mm [36]. We predicted that since TA has anti-QS properties, it may suppress the development of pathogenic *P. aeruginosa* or its virulence components that are regulated by QS. Interestingly, tannic acid exhibited broad spectrum antibacterial activity against selected isolates of XDR *P. aeruginosa*, showing highest activity against PA-05. The results aligned with Strompfová et al. [37], who reported that TA showed the highest antibacterial activity among eight plant-derived bioactive compounds. Moreover, Reyes et al. [38] studied the protective effect of TA (80 µg/mL) against *Salmonella enterica* infection in mice without showing cytotoxic effect. This indicates that TA has a potential to be a safe antibacterial agent. However, development of a new TA-based treatment for *P. aeruginosa* infections requires more studies using animal models. 

*P. aeruginosa* has three QS networks (Las, Rhl and Pqs) which control the genomic expression of several virulence traits. BCL was previously evaluated at sub-inhibitory concentrations for QS and biofilm inhibition properties against *P. aeruginosa* [38]. Our study demonstrated that TA has a similar inhibitory impact on QS systems as BCL, which was supported by in vitro and in silico investigations [39]. According to our results, the production of pyocyanin was inhibited by TA. Pyocyanin is a vital component of *P. aeruginosa* infections; it is a redox-active toxin and a noticeable green-colored phenazine pigment. Rhl, together with RhlR and the autoinducer signal molecule C4-HSL, is responsible for promoting the expression of pyocyanin production in *P. aeruginosa* via the QS system [40]. This reduction in pyocyanin pigment by TA (89%) was greater than that achieved in a previous study, in which 0.75 mg/mL ethyl acetate extract, ethanolic extract, and N-butanol extract of Chi flower (*Camellia nitidissima*) decreased pyocyanin production by 56.9%, 51.2%, and 51.5%, respectively [41].

**Figure 8:**
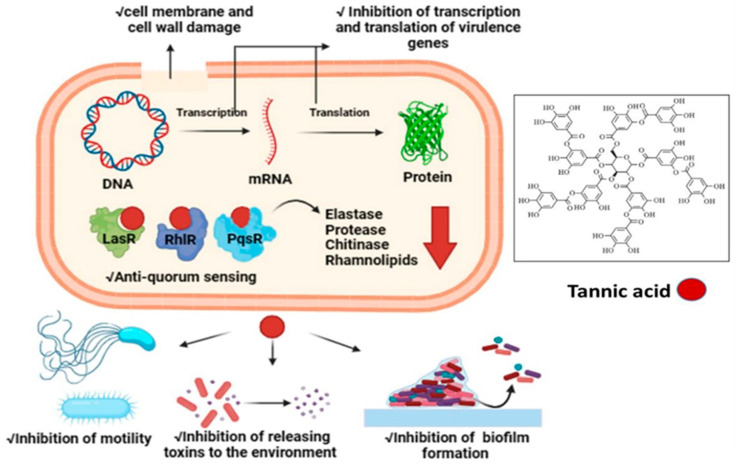
A proposed mechanism of antibacterial and anti-QS actions of tannic acid against *P. aeruginosa*.

Elastases and proteases initiate the first development of infection in host tissues. *P. aeruginosa* releases various protease and elastase virulence factors that are controlled by LasI/R, indicating that they have a role in pathogenicity [42]. The gene *lasB* encodes elastase, a strong T2SS-secreted proteolytic enzyme. It has a diverse spectrum of substrates, including a wide range of connective tissue components, such as collagen, elastin, and fibronectin. In order to facilitate pathogen invasion, these bacterial proteases serve as hydrolytic enzymes that specifically target host proteins [43]. Moreover, the activity of bacteria that can break down chitin, known as chitinolytic activity, is crucial for the recycling of carbon and nitrogen sources into a readily available form in the environment [44]. Chitinase enzyme expression increases in clinical isolates, perhaps contributing to severe infection [45]. The impact of TA on LasA protease, LasB elastase, and chitinase in our study suggested that TA has the potential to limit the development of acute infections in the host, since TA suppressed the production of all virulence features of PA-05 when compared to the control. Interestingly, comparable findings were found in the study of Zhong et al. [46]. Catechin-7-Xyloside, sappanol, and butein were shown to inhibit the development of virulence factors and pathogenesis in *P. aeruginosa*, such as protease and elastase [46]. In another study, Husain et al. [47] observed 80% decrease in chitin synthesis when *P. aeruginosa* was treated with 1.6% of clove oil. Furthermore, *P. aeruginosa* motility is important in biofilm surface adhesion and development [47]. *P. aeruginosa* can swim and swarm on liquid and semisolid substrates because to its polar flagellum [48,49]. TA inhibited the motility of PA-05 more effectively than BLC. According to previous research, aspirin inhibited the swimming motility of *P. aeruginosa* by 34% [50]. Similarly, Liu et al. [51] observed that at a concentration of 1 g/mL, cinnamaldehyde may inhibit swarming by up to 58.4% and swimming by up to 40.7%. In comparison to previous studies, we were able to obtain higher inhibition of swimming and swarming motility of PA-05 by TA. Furthermore, phospholipids of lung surfactant are known to be more vulnerable to phospholipase C breakage, whereas rhamnolipid, a biosurfactant composed of a glycolipid detergent-like structure containing rhamnose, is known to solubilize these phospholipids [52]. Our results showed a greater reduction in rhamnolipid formation (88% vs. 19.03% and 21.61%, respectively) when treated with betulin and betulinic acid at 125 g/mL doses, beyond previous studies [53]. We predicted that since TA substantially suppresses QS and virulence factors production in PA-05, it could also reduce the expression of QS-related genes. The expression levels of QS-associated genes, including *lasI*, *lasR*, *rhlR*, and *rhlI*, were measured in PA-05 cells treated with TA by quantitative RT-PCR. Our results showed that TA dramatically reduced the mRNA levels of QS proteins (*lasR*, *lasI*, *rhlR*, *rhlI, pqsR*, and *pqsA*) in PA-05. Our findings suggested that TA suppresses QS via suppressing the expression of QS-related genes similar to those obtained by Mishra [9]. Molecular docking is an effective approach that can be used to estimate binding affinities and drug option bound structures [54,55,56]. For the first time, the molecular interaction of TA with *P. aeruginosa* QS receptors was examined using molecular docking. For each QS system, the activation of the receptor protein (LasR, RhlR, and PasR) by the relevant signal molecules generates additional stimulation molecules, which are also responsible for the up-regulation of different genes related to virulence, secondary metabolites, and biofilm production. Therefore, the docking data revealed a competitive binding of TA with LasR, RhlR, and PqsR proteins. Anju et al. recorded lower binding affinity of BCL and autoinducers (N-3-oxo-dodecanoyl homoserine lactone and butanoyl homoserine lactone) with LasR and RhlR [56]. Thus, the obtained data suggest that TA from *P. oxalicum* can act as a promising anti quorum agent with antibacterial properties against XDR *P. aeruginosa*. Figure 8 explains the antibacterial and anti-QS mechanism of action of TA.

## 4. Materials and Methods

### 4.1. Isolation of Endophytes and Screening for Their Anti-QS Activity

In the present study, we used ten endophytic fungal strains collected in 2021/2022, as described in our previous work [1]. These strains were labeled as EF1-EF10. They were isolated from cladodes of *Opuntia ficus-indica* (L.) Mill. (Cactaceae) placed on agar plates with potato dextrose medium (Sigma-Aldrich, St. Louis, MO, USA) supplemented with penicillin (100 U/mL) and then incubated at 30 °C for 1 week. Then, the purity of the cultures was checked to ensure and preserve their purity as axenic cultures. Endophytic fungi were grown separately on potato dextrose broth at 30 °C for 14 days. Then fungal filtrates were collected and tested for their anti-QS activities on the *C. violaceum* monitor strain (ATCC 12472) using the agar-well diffusion procedure. *C. violaceum* was equally spread on the Luria–Bertani agar plates (Hi-Media, Mumbai, MH, India), and then 100 µL of each fungal crude sample was added in each well. After incubating the plates at 37 °C for 24 h, the diameter of colorless halo zones surrounding the wells was measured to determine the inhibitory activity of violacein pigment production [9]. As a negative control, we used 1% dimethyl sulfoxide (DMSO) and 250 µg/mL baicalein (BCL) as a positive control (both compounds manufactured by Sigma-Aldrich, St Louis, MO, USA). The isolate with the largest clear zone diameter was chosen as the most potent and utilized for the subsequent investigation, which included obtaining and purifying the QS inhibitor compound. 

### 4.2. Identification of the Chosen Fungal Species

Isolate EF10 was chosen for further work after screening all isolates. The morphology of hyphae and conidia was used for microscopic identification of the selected fungus [57]. In order to confirm the species identification of the selected endophytic fungal isolate using molecular techniques, we sent culture samples to the Molecular Biology Research Unit at Assiut University (AUMC), where fungal DNA was extracted with a Patho Gene-spin^TM^ DNA/RNA extraction kit (Intron Biotechnology, Seongnam-Si, GG, Republic of Korea). Then, the nuclear ribosomal internal transcribed spacer (ITS) region was amplified using the primers ITS1 (5′-TCC GTA GGT GAA CCTGCG G-3′) and ITS4 (5′-TCC TCC GCT TAT TGA TAT GC-3′) [58] and sequenced using capillary sequencing, followed by phylogenetic analysis carried out using MEGA X software 11.0.10, as described in White et al. [59]. 

### 4.3. Obtaining a Crude Extract Form the Culture of the Chosen Fungal Isolate

The endophytic fungal isolate EF10 was chosen for the extraction of the active compounds because it showed the strongest anti-QS activity. The chosen isolate was grown in potato dextrose broth at 30 °C for 14 days. Following fermentation, the extracellular secondary metabolites were obtained with ethyl acetate as extracting solvent [60]. Once the broth culture was placed in a funnel, the mycelia were extracted by filtering it using Whatman No. 1 filter paper. After extracting the filtrate three times with the same volume of ethyl acetate, it was left to stand for 20 min. Finally, the organic solvent phase was concentrated using a rotary evaporator by separating it at low pressure [58]. After that, the crude extract (1 mg/mL) was stored at 20 °C until further use (Figure 1). 

### 4.4. Fractionation and Purification of the Active Compound

Column chromatography was used for further fractionation of the concentrated crude sample of the selected isolate EF10. Silica gel with a mesh size of 60–120 (Merck, Darmstadt, Germany) served as the stationary phase. It was packed onto a glass column of 700 × 30 mm. The sample bed consisted of a mixture of crude extract (dried powdered) and silica gel/powder (200 mesh size), with the ratio being 1:3. The column was first eluted with hexane, then with an increasing polarity combination of hexane and ethyl acetate (9:1, 8:2, 7:3, 6:4, 5:5, etc.), and then finally with pure ethyl acetate, methanol, and water. The anti-QS activity of the eight fractions (F1-F8) was evaluated [2]. Then, the active fraction (F5) was analyzed by thin layer chromatography (TLC) to confirm purification. The stationary phase was a silica gel G60 F254 plate (Merck, Darmstadt, Germany). Formic acid, ethyl acetate, and toluene (in a ratio of 1:4:5) were used as the mobile phase [61]. The technique of separation was carried out in accordance with El-Zawawy et al. [62]. The retention factor (R_f_) value of the active fraction was determined after the separation procedure. Then, the band was scratched from TLC and kept for further characterization steps (Figure 1). 

### 4.5. Characterization and Identification of the Active Compound

In order to identify the functional group in the isolated active compound, Fourier transform infrared spectroscopy (FTIR) was performed using a Thermo Nicolet model 6700 IR source with a spectrum range of 500–4000 cm^−1^. Moreover, many different vibrational modes have been observed using FT-Raman spectroscopy by means of RFS 27 spectrometer (Bruker, Billerica, MA, USA) [63]. Nuclear magnetic resonance (NMR) spectroscopy utilizing a Bruker Avance II 400 (US) spectrometer was used to determine the structure of the isolated active compound [64]. Additionally, in order to confirm the identification of the isolated active compound as tannic acid (TA) compared to standard tannic acid (Sigma-Aldrich, Saint Louis, MO, USA), high-performance liquid chromatography (HPLC) using Dionex Ultimate 3000 UHPLC apparatus (Thermo Scientific, Waltham, MA, USA) was carried out at Pharmaceutical Service Center, Faculty of Pharmacy, Tanta University, Egypt [65]. 

### 4.6. Antibacterial Activity of the Isolated Active Compound (TA) 

Fourteen isolates of XDR *P. aeruginosa* were previously obtained from infected human lungs [66]. These isolates were named PA-01 to PA-14. The antibacterial activity of TA and standard tannic acid (Sigma-Aldrich) against these isolates was determined separately using the agar-well diffusion technique, as previously described by El Zawawy et al. [67]. Each bacterial culture suspension (10^6^ CFU/mL) was prepared, and the Mueller–Hinton agar plates were inoculated with 100 µL of each isolate broth culture. TA was dissolved to a final concentration of 1000 µg/mL (in 1% DMSO). One hundred microliters of TA (125, 250, 500, and 1000 µg/mL) were pipetted into a well (with 5 mm diameter) formed in the middle of the agar plate. Streptomycin (10 µL) and DMSO (1%) were used as positive and negative controls, respectively [68]. Furthermore, the effect of TA on bacterial cells was visualized by transmission electron microscopy (TEM) using JEM-100SX microscope (JEOL, Japan) at Faculty of Medicine, Tanta University, Tanta, Egypt. 

### 4.7. Anti-QS Potential of the Isolated TA 

TA was subsequently assessed for anti-QS activity against PA-05 (the most susceptible isolate to TA), as described below, in comparison to BLC, a compound with the ability to reduce *P. aeruginosa* virulence factors by down regulating genes regulated by QS, which was used as a positive control [41]. As a negative control, 1% DMSO was utilized. PA-05 was cultured in LB broth and then incubated in the presence of TA or BLC to obtain MICs and sub-MICs to perform further experiments. 

### 4.8. Assessment of Minimal Inhibitory Concentrations (MICs) and Growth Curves

The MIC of TA and BCL was measured against *P. aeruginosa* isolate PA-05 using the microdilution technique with an inoculum of 10^6^ CFU/mL based on Clinical and Laboratory Standards Institute criteria [69]. The MIC of each selected compound was determined as the lowest concentration that stopped observable growth. The PA-05 inoculum from overnight cultures were diluted in freshly made LB medium to achieve a cell-suspension of 0.05 optical density at 600 nm (OD_600_) for measuring the growth curve. The suspensions were then added to various concentrations of TA (100, 75, and 45 µg/mL) and BCL (200, 175, and 150 µg/mL) and incubated at 37 °C. The turbidity at OD_600_ was then measured spectrophotometrically (Thermo Fisher Scientific, Waltham, MA, USA) and recorded to create a growth curve. The sub-MICs were determined as concentrations equivalent to or lower than the greatest TA and BCL concentrations that did not impair growth. Every experiment was carried out in three replicates following methods of Bala et al. [70]. 

### 4.9. Efficacy of TA on Virulence Factors Regulated by QS 

PA-05 culture supernatants were prepared to quantify several QS-regulated virulence factors. Overnight bacterial cultures were kept for 24 h at 37 °C with the sub-MICs of TA and BCL and without as a negative control (untreated bacterial culture) for the assays specified below. 

#### 4.9.1. Pyocyanin Production Assay

The inhibition of pyocyanin pigment formation was assessed quantitatively. In brief, 1 mL of each PA-05 cell-free culture supernatant was extracted with an equivalent proportion of chloroform. After extraction, 1 mL of 0.2 N HCl was used to remove the organic phase, and the quantity of pyocyanin was detected spectrophotometrically at 520 nm [71].

#### 4.9.2. Chitinase Activity Assay

The inhibition of chitinase activity was determined using a modified chitin-azure test [49]. As a substrate, 0.5 mg/mL of chitin azure was utilized and liquefied in sodium citrate buffer (0.1 M, pH 4.8). Briefly, one milliliter of each cell-free supernatant of PA-05 was incubated with 0.5 mL of substrate solution for 7 days at 37 °C with continual agitation (150 rpm). After centrifugation at 10,000 rpm to remove the insoluble substrate, the absorbance of the obtained supernatant was measured at 570 nm.

#### 4.9.3. LasA Protease Assay

The proteolytic activity of PA-05 was assessed using the method described by Hentzer et al. [72], with minor modifications. For 30 min at 37 °C, a volume of 500 µL of substrate solution and about 1 mL 0.3% azocasein (prepared in a solution of 50 mM Tris at pH 7.8) were combined with 100 µL of each cell-free supernatant of PA-05. After that, 0.5 mL of prechilled 10% trichloroacetic acid was then added and allowed to incubate at 4 °C for 15 min. This step was carried out to allow the undigested substrate to form a precipitate. Then, protease activity was measured as the absorbance at 400 nm of supernatants obtained after 10,000 rpm centrifugation. 

#### 4.9.4. LasB Elastase Assay 

According to Ohman et al. [73], the elastolytic activity of each cell-free supernatant of PA-05 was tested. Briefly, 100 microliters of each PA-05 culture supernatant was mixed with 900 µL of elastin congo red buffer (20 mg of elastin congo red, 100 mM Tris, 1 mM CaCl2, pH 7.5). Then, the resultant mixture was kept for 3 h at 37 °C. Subsequently, the elastolytic activity was assessed by detecting the absorbance at 495 nm of the supernatants collected following 10 min centrifugation at 10,000 rpm.

#### 4.9.5. Rhamnolipids Assay

The quantitation rhamnolipids followed the protocol from Luo et al. [74]. Each PA-05 cell-free culture supernatant was dried after being extracted with twice the amount of ethyl acetate. The dried mixture was mixed back into the orcinol solution, including 900 µL of 0.19% orcinol dissolved in 53% *v*/*v* sulfuric acid. Then, the samples were measured spectrophotometrically at 421 nm after being incubated at 80 °C for 30 min. 

### 4.10. Motility Behavior of PA-05

The swimming and swarming motilities of PA-05 after treatment with TA or BCL were compared to untreated control using previously reported techniques [75]. For the swimming experiment, a sterile toothpick was used to inject 100 µL of each PA-05 culture supernatant into the middle of a 5 mm LB medium plate. The plate contained 0.2% (*w*/*v*) casamino acids, 0.3% (*w*/*v*) Bacto agar, and 30 mM glucose. Following 24 h incubation at a temperature of 37 °C, the swimming zone was assessed. Bacteria were inserted into the middle of swarm plates with 0.4% (*w*/*v*) Bacto agar, LB with 0.5% (*w*/*v*) casamino acids, and 0.5% (*w*/*v*) glucose using sterile toothpicks in order to assess swarming motility. Subsequently, the plates were examined after 24 h of incubation at 37 °C. 

### 4.11. Extraction of RNA and Measurement of Gene Expression Using Quantitative Real-Time PCR (qRT-PCR)

Total RNA was extracted from each PA-05 cell-free culture supernatant using an RNeasy Mini Kit (Qiagen NV, Venlo, The Netherlands) and quantified using a spectrophotometer (NanoDrop 2000c; Thermo Fisher Scientific) for qRT-PCR analysis. The complementary DNA was synthesized in reaction volume of 20 μL using the High-Capacity cDNA Reverse Transcriptase Kit (Applied Biosystems, Thermo Fisher Scientific). Then, 1 μg of cDNA was used to amplify the PA-05 genes *pelF*, *pslA*, *rhlR*, *lasR*, *pqsR*, *lasI*, *rhlI*, *pqsA*, *toxA*, *exoS*, and *aprA* by means of ViiA7 real-time PCR equipment (Applied Biosystems). Primers were designed by GenScript based on the cDNA sequences for each gene, according to Liu et al. [76] and Anju et al. [77], as shown in Table S1. All results were normalized with respect to the housekeeping gene (*proC*) to determine the relative changes in gene expression. Each real-time PCR experiment was run in triplicate on three different cultures. 

### 4.12. Molecular Docking

To evaluate binding affinity, three-dimensional (3D) structure of TA (ligand) was docked to 3D structures of QS receptor proteins. The 3D structure of LasR protein (PDB ID: 2UV0), RhlR (ID: 8B4A), and PqsR protein (ID: 6Q7U) was obtained from the RCSB-Protein Data Bank. Also, the 3D structure of TA (ID:DB09372) was retrieved from drug bank, and optimized with the aid of Avogadro software 1.2.0. Then AutodockTools 1.5.7 software [78] was used to detect the active site of selected proteins and to prepare them through removing all water molecules and heteroatoms, while polar hydrogen and Kollman charges were added in addition to repairing missing atoms. The coordinates of proteins were saved in pdbqt format for docking purposes. The dimensions of the GRID box were selected to cover all of the residues within the active site. The grid spacing was set to 0.375 Å. The docking procedure was executed via Autodock Vina [79]. Analysis of the docking result was conducted using Biovia Discovery Studio v21.1.0.20298 

### 4.13. Statistical Analysis 

GraphPad Prism 8.0 Software (San Diego, CA, USA) was used to analyze the data. Each experiment was replicated three times. One-way ANOVA analyses with Tukey’s multiple comparison tests or two-way ANOVA followed by Dunnett’s or Sidak’s multiple comparison tests were performed to determine statistical difference between samples.

## 5. Conclusions

Endophytes have been considered to be a rich source of naturally occurring bioactive compounds. This is the first study in which TA has been isolated from the endophytic fungus *P. oxalicum* from the cladodes of *Opuntia ficus-indica*. The TA compound shows high antibacterial activity against the XDR *P. aeruginosa*, and it has potential as an anti-QS compound using several assays and investigations. Moreover, our research reveals that TA inhibits the biosynthesis and genomic expression of important QS-controlled virulence characteristics and biofilm determinants. Furthermore, the mechanism of action of TA was explained using in silico analysis, which revealed that TA had a strong interaction with LasR, RhlR and PqsR proteins. Our research highlights the potential of *P. oxalicum* as an alternate source of TA as a QS inhibitor for treating *Pseudomonas* infections that are resistant to traditional anti-bacterial treatment. Further investigations using animal models are required to validate the effectiveness of TA’s antibacterial properties.

## Figures and Tables

**Table 1:** Anti-QS activity of extracts from fungal endophytes isolated from *Opuntia ficus-indica* using well diffusion method and a monitor strain of *Chromobacterium violaceum*. Diameters of inhibition zones were measured around wells with extracts from cultures of fungal isolates in three replicates. Values are means ± standard deviation ^1^.

Fungal Extract	Diameter Zone (mm)
EF1	17 ^bc^ ± 0.6
EF2	18 ^bc^ ± 0.1
EF3	10 ^e^ ± 0.1
EF4	19 ^b^ ± 0.1
EF5	14 ^d^ ± 0.0
EF6	16 ^c^ ± 0.6
EF7	0 ^f^
EF8	0 ^f^
EF9	19 ^b^ ± 0.3
EF10	23 ^a^ ± 0.0
Positive control (baicalein)	18 ^bc^ ±0.0

^1^ Different letters next to mean values indicate statistically significant differences (*p* < 0.0001).

**Table 2:** Antibacterial activity of tannic acid tested using well diffusion method against 14 isolates of extensively drug-resistant *P. aeruginosa*. Values are means ± standard deviation ^1^.

Isolate Code	Diameter of the Inhibition Zone (mm) in a Test with Tannic Acid Applied in the Following Concentrations:			
	125 µg/mL	250 µg/mL	500 µg/mL	1000 µg/mL
PA-01	6 ^f^ ± 0.0	14 ^f^ ± 0.1	23 ^e^ ± 0.6	30 ^e^ ± 0.0
PA-02	7 ^e^ ± 0.2	16 ^d^ ± 0.0	26 ^c^ ± 0.0	33 ^b^ ± 0.1
PA-03	11 ^a^ ± 0.1	18 ^b^ ± 0.0	27 ^b^ ± 0.3	33 ^b^ ± 0.1
PA-04	7 ^e^ ± 0.3	14 ^f^ ± 0.1	21 ^h^ ± 0.0	30 ^e^ ± 0.0
PA-05	12 ^a^ ± 0.3	19 ^a^ ± 0.6	28 ^a^ ± 0.0	35 ^a^ ± 0.3
PA-06	8 ^d^ ± 0.0	15 ^c^ ± 0.7	24 ^e^ ± 0.1	31 ^d^ ± 0.0
PA-07	7 ^e^ ± 0.0	13 ^g^ ± 0.0	24 ^d^ ± 0.6	31 ^d^ ± 0.1
PA-08	7 ^e^ ± 0.5	15 ^d^ ± 0.9	22 ^f^ ± 0.1	29 ^f^ ± 0.0
PA-09	7 ^e^ ± 0.3	15 ^e^ ± 0.5	21 ^g^ ± 0.0	30 ^e^ ± 0.5
PA-10	8 ^d^ ± 0.3	16 ^d^ ± 0.5	23 ^f^ ± 0.0	33 ^b^ ± 0.1
PA-11	9 ^c^ ± 0.0	16 ^d^ ± 0.0	22 ^g^ ± 0.1	30 ^e^ ± 0.0
PA-12	9 ^c^ ± 0.1	16 ^c^ ± 0.9	21 ^g^ ± 0.9	30 ^e^ ± 0.7
PA-13	8 ^d^ ± 0.1	15 ^e^ ± 0.2	22 ^f^ ± 0.3	29 ^f^ ± 0.5
PA-14	10 ^b^ ± 0.0	17 ^c^ ± 0.3	25 ^d^ ± 0.0	32 ^c^ ± 0.5

^1^ Different letters (in the same column) next to mean values indicate statistically significant differences (*p* < 0.0001).

## Data Availability

All other relevant data are included in the manuscript and its Supplementary Materials.

## Supplementary Materials

The following supporting information can be downloaded at: https://www.mdpi.com/article/10.3390/ijms252011115/s1.
