# Supplementary material for: Endophytic Penicillium oxalicum AUMC 14898 from Opuntia ficus-indica: A Novel Source of Tannic Acid Inhibiting Virulence and Quorum Sensing of Extensively Drug-Resistant Pseudomonas aeruginosa
Source: Int J Mol Sci. 2024 Oct 16;25(20):11115. doi: 10.3390/ijms252011115 (PMC11507641; doi:10.3390/ijms252011115)
Supplement: Supplementary file 1 [file ijms-25-11115-s001.zip › ijms-3236837-supplementary.pdf]

Supplementary materials

Endophytic *Penicillium oxalicum* AUMC 14898 from *Opuntia ficus-indica*: A Novel Source of Tannic Acid Inhibiting Virulence and Quorum Sensing of Extensively Drug-Resistant *Pseudomonas aeruginosa*

Hoda S. Nouh[1*], Nessma A. El-Zawawy[1], Mohamed Halawa[1], Ebrahim M. Shalamesh[1], Sameh Samir Ali[1], Grażyna Korbecka-Glinka[2*], Awad Y. Shala[3], Shimaa El-Sapagh[1]

[1] Botany and Microbiology Department, Faculty of Science, Tanta University, 31511 Tanta, Egypt
[2] Department of Biotechnology and Plant Breeding, Institute of Soil Science and Plant Cultivation – State Research Institute, 24-100 Puławy, Poland
[3] Medicinal and Aromatic Plants Research Dept., Horticulture Research Institute, Agricultural Research Center, 12619 Giza, Egypt
* Correspondence: hoda.nouh@science.tanta.edu.eg (HSN); gkorbecka@iung.pulawy.pl (GKG)

Table S1. Primers used for *P. aeruginosa* in qRT-PCR study

	Gene	Gene ID	Forward primer	Reverse primer
1	*proC* (Reference gene)	878413	CAGGCCGGGCAGTTGCTGTC	GGTCAGGCGCGAGGCTGTCT
2	*lasR*	881789	ACGCTCAAGTGGAAAATTGG	TCGTAGTCCTGGCTGTCCTT
3	*lasI*	881777	GGCTGGGACGTTAGTGTCAT	AAAACCTGGGCTTCAGGAGT
4	*rhlI*	878967	AAGGACGTCTTCGCCTACCT	GCAGGCTGGACCAGAATATC
5	*rhlR*	878968	CATCCGATGCTGATGTCCAACC	ATGATGGCGATTTCCCCGGAC
6	*lasB*	880368	GACCGAGAATGACAAAGTGGAA	GGTAGGAGACGTTGTAGACCGT
7	*aprA*	881248	CCCTGTCCTATTCGTTCCTG	GCGTCGACGAAGTGGATATT
8	*toxA*	877850	GGAGCGCAACTATCCCACT	TGGTAGCCGACGAACACATA
9	*exoS*	879837	GGCGGATGCGGAAAAGTAC	CTGACGCAGAGCGCGATT
10	*pelF*	879152	GCCGATGAAGAACACCGAGAAGG	CCGATGTCTGCCTGCTGCTG
11	*pslA*	879717	GCCGCTTCATCCGCAAGACC	TGTATTCGCTGACCGCCTCCTC
12	*pqsR*	879994	CTGATCTGCCGGTAATTGG	ATCGACGAGGAACTGAAGA
13	*pqsA*	880760	GAAGTGAGCGAGGCGGTTCTG	CTGTTCGGCGAGATGCTGGTC

Table S2. Anti-QS activity of the obtained fractions from EF10 isolate. The diameter zone values are means from three replicates ± standard deviation[1].

Fractions	Anti-QS activity
	Diameter zone (mm)
**F1**	0^{e}
**F2**	0^{e}
**F3**	$11^{c}\pm 0.0$
**F4**	0^{e}
**F5**	$25^{a}\pm 0.3$
**F6**	$14^{b}\pm 0.4$
**F7**	$10^{c}\pm 0.4$
**F8**	$8^{d}\pm 0.0$

[1] Different letters next to the mean values indicate statistically significant differences ($p<0.0001$)

Table S3. Antibacterial activity of standard tannic acid (1000 μg/mL). The diameter zone values are means from three replicates ± standard deviation[1].

Isolate code	Diameter zone (mm)
PA-01	$32^{b}\pm 0.0$
PA-02	$30^{c}\pm 0.0$
PA-03	$32^{b}\pm 0.2$
PA-04	$32^{b}\pm 0.1$
PA-05	$33^{a}\pm 0.0$
PA-06	$31^{b}\pm 0.0$
PA-07	$28^{d}\pm 0.0$
PA-08	$31^{b}\pm 0.0$
PA-09	$30^{c}\pm 0.2$
PA-10	$30^{c}\pm 0.1$
PA-11	$29^{d}\pm 0.0$
PA-12	$32^{b}\pm 0.2$
PA-13	$28^{d}\pm 0.1$
PA-14	$32^{b}\pm 0.0$

[1] Different letters next to the mean values indicate statistically significant differences ($p<0.0001$)

Table S4. Molecular docking of TA with LasR, RhlR and PqsR receptors showing docking score, hydrogen bonds, interacting residues and bond length.

Receptors	Docking score (kcal.mol^{-1})	Number interactions	H-bonds	H-bond length (Å)
**LasR**	-9.5	7	TYR69<br>GLN81<br>THR95<br>LYS97<br>GLN98<br>ARG71<br>TYR47	2.4<br>2.2<br>2.4<br>2.6<br>2.0<br>3.4<br>2.2
**RhlR**	-9.2	4	HIS61<br>LYS66<br>GLN73<br>VAL6	2.2<br>2.8<br>2.7<br>3.3
**PqsR**	-11	4	GLN194<br>LEU197<br>ASN20<br>ALA187	2.8<br>2.2<br>2.2<br>3.2

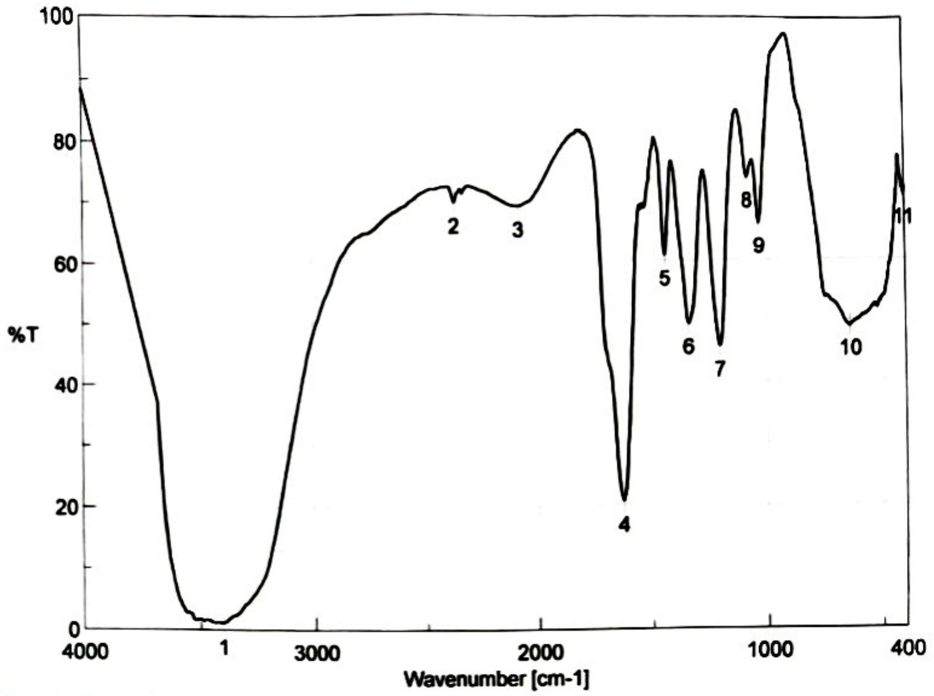

Figure S1. FT-IR spectra of standard tannic acid.

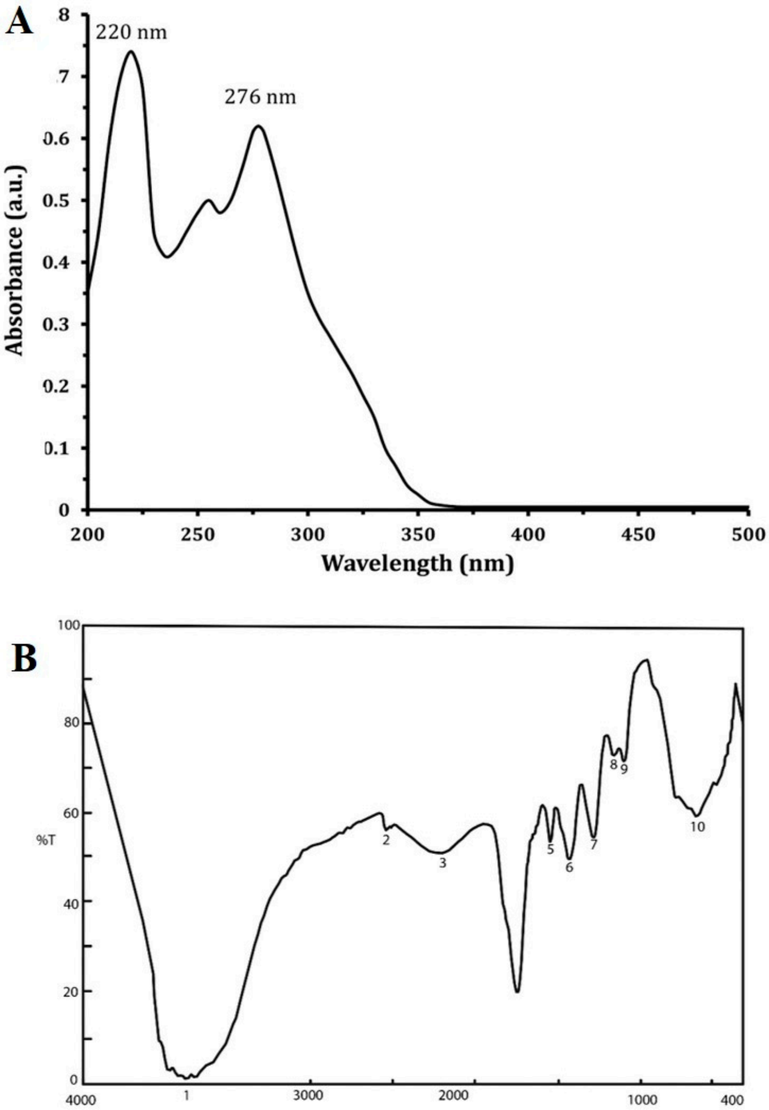

Figure S2. (A) UV-visible and(B) FTIR spectra of the compound purified from *Penicillium oxalicum* AUMC 14898.

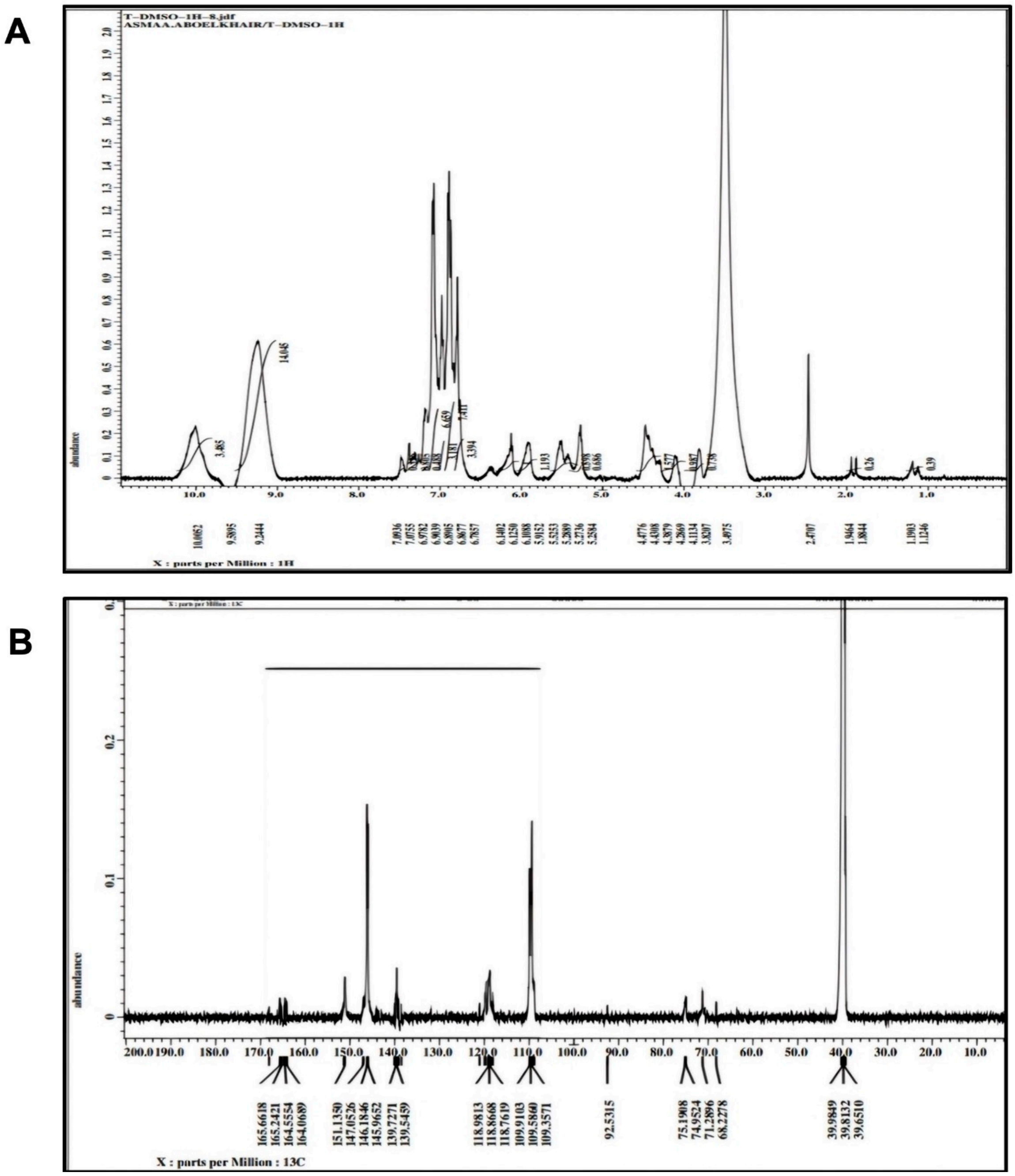

Figure S3. ^{1}H NMR (A) and ^{13}C NMR (B) spectra of standard tannic acid.

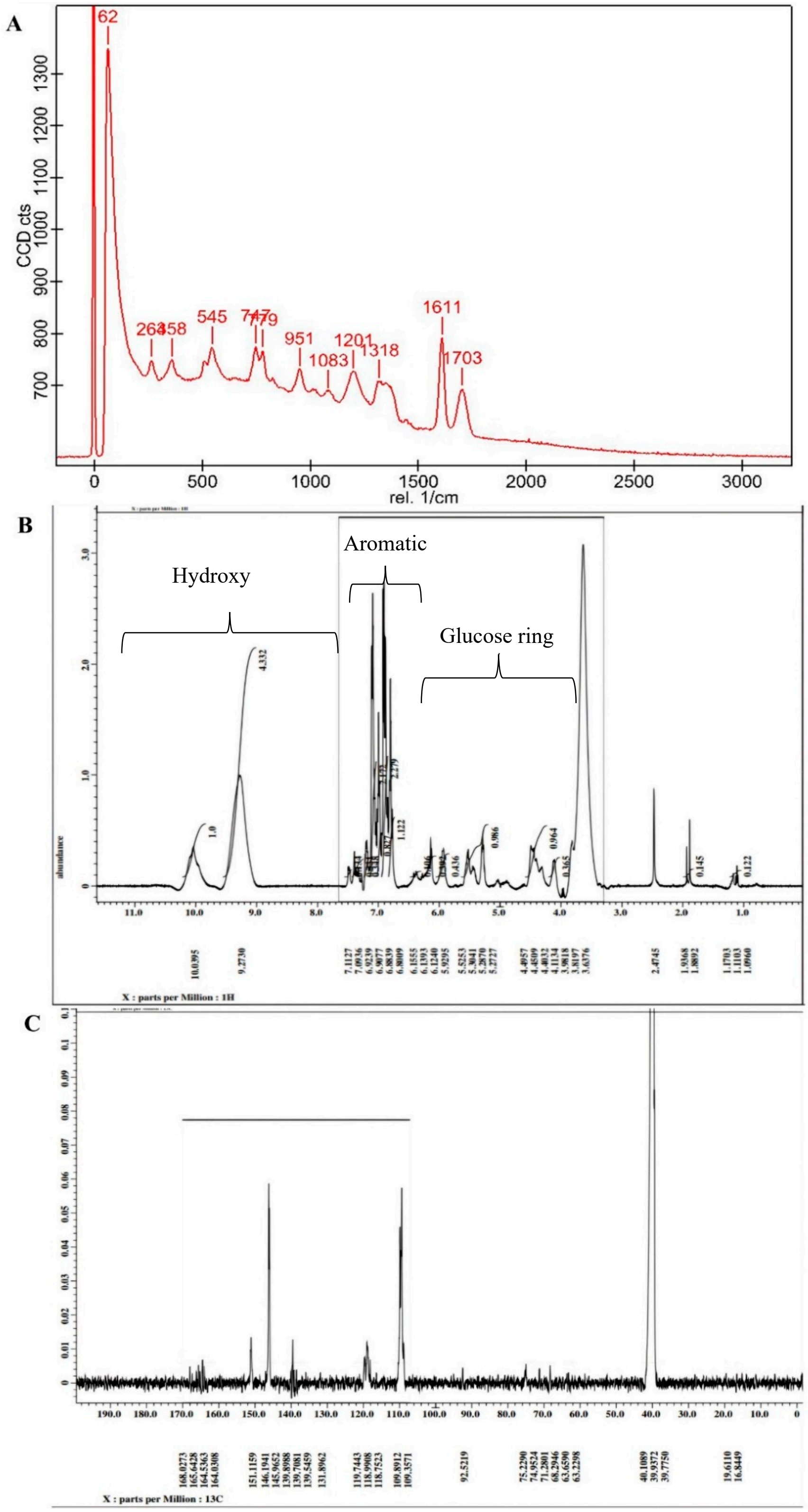

Figure S4. FT-Raman (A), ^{1}H NMR (B) and ^{13}C NMR (C) spectra of purified compound from *Penicillium oxalicum* AUMC 14898, the spectra indicated the aromatic structure of tannic acid. Raman spectrum showed wide spectral bands with two strong peaks at 1703 and 1611 cm^{-1} which are characteristic of tannic acid, while NMR spectra represented carbon and hydrogen content of the compound indicating its aromatic nature.

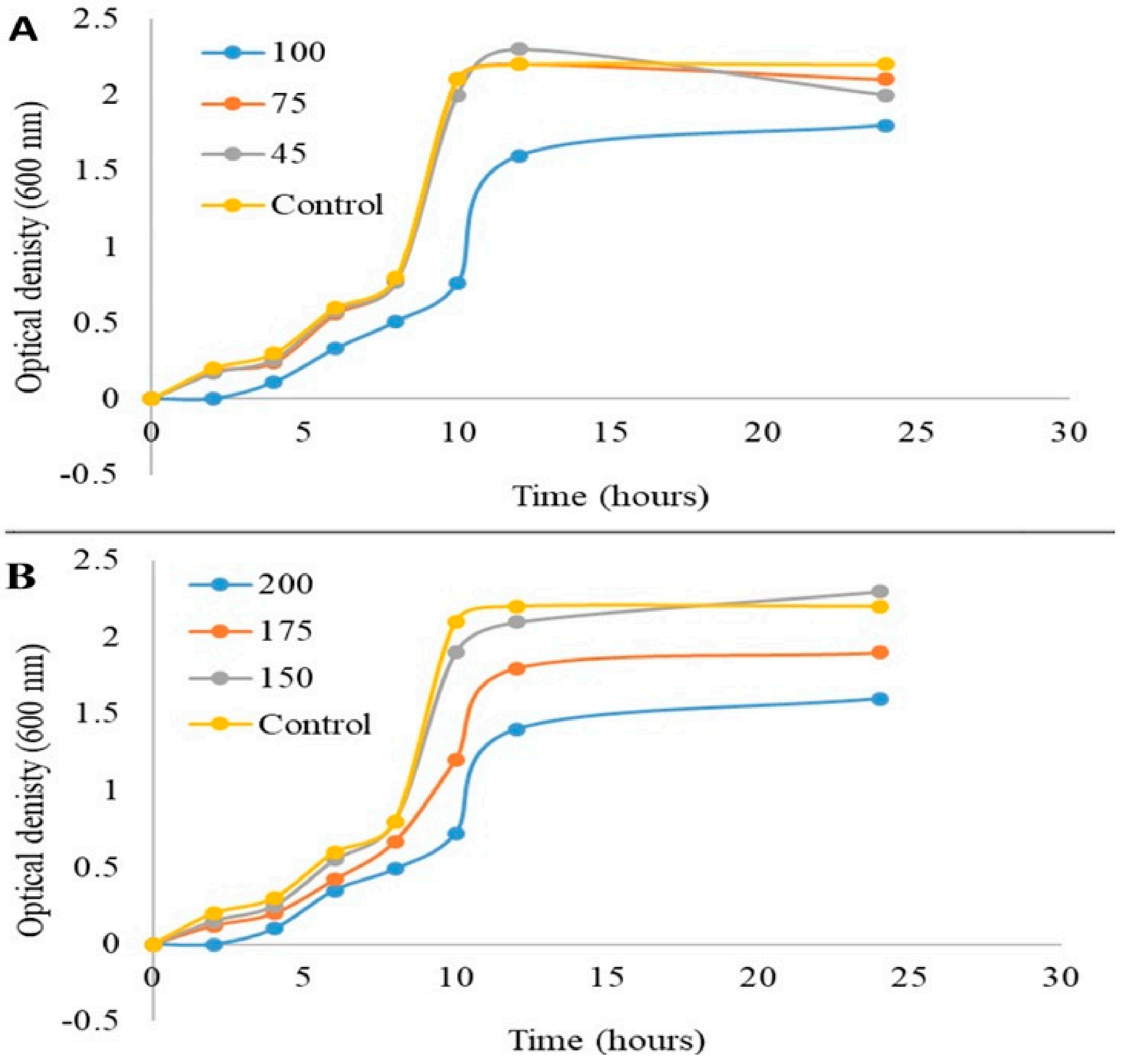

Figure S5. Antibacterial effect of tannic acid (TA) and baicalein (BCL) against *P. aeruginosa* isolate PA-05: **(A)** Growth curves of the PA-05 strain in control and at sub-MICs (45-100 µg/mL) of TA. **(B)** Growth curves of the PA-05 strain in control and at sub-MICs (150-200 µg/mL) of BCL.

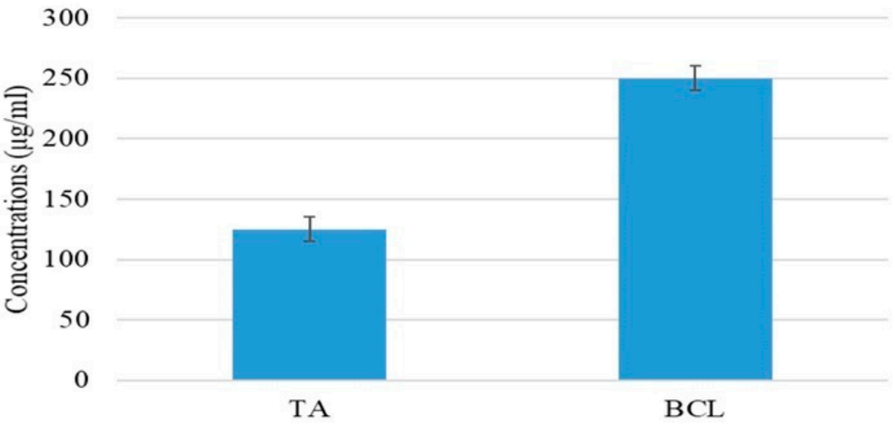

Figure S6. Minimum inhibitory concentrations of tannic acid (TA) and baicalein (BCL) against *P. aeruginosa* isolate PA-05. Presented values are averaged over three replicates ± standard error
